# Group Movement in Entomopathogenic Nematodes: Aggregation Levels Vary Based on Context

**DOI:** 10.2478/jofnem-2024-0002

**Published:** 2024-03-14

**Authors:** Glen Stevens, Muhammad Usman, Sehrish Gulzar, Cassandra Stevens, Eleanor Pimentel, Hilal Erdogan, Paul Schliekelman, Fatma Kaplan, Hans Alborn, Waqas Wakil, David Shapiro-Ilan, Edwin E Lewis

**Affiliations:** University of Idaho, Department of Entomology, Plant Pathology and Nematology, Moscow, ID 83844, USA; Department of Entomology, University of Agriculture Faisalabad 38000, Pakistan; Faculty of Agriculture, Department of Biosystems Engineering, Bursa Uludağ University, Bursa 16059, Turkey; University of Georgia, Department of Statistics, Athens, GA 30602, USA; Pheronym, Inc., Davis, CA 95618, USA; Center for Medical, Agricultural, and Veterinary Entomology, U.S. Department of Agriculture Agricultural Research Service, 1700SW 23rd Drive, Gainesville, FL, USA; USDA-ARS, SEFTNRL, Byron, GA 31008, USA

**Keywords:** behavior, entomopathogenic nematode, group behavior, Index of Dispersion

## Abstract

Maintenance of an aggregated population structure implies within-species communication. In mixed-species environments, species-specific aggregations may reduce interspecific competition and promote coexistence. We studied whether movement and aggregation behavior of three entomopathogenic nematode species changed when isolated, as compared to mixed-species arenas. Movement and aggregation of *Steinernema carpocapsae*, *S. feltiae* and *S. glaseri* were assessed in sand. Each species demonstrated significant aggregation when alone. Mixed-species trials involved adding two species of nematodes, either combined in the center of the arena or at separate corners. While individual species became less aggregated than in single-species conditions when co-applied in the same location, they became more aggregated when applied in separate corners. This increased aggregation in separate-corner trials occurred even though the nematodes moved just as far when mixed together as they did when alone. These findings suggest that maintenance of multiple species within the same habitat is driven, at least in part, by species-specific signals that promote conspecific aggregation, and when the species are mixed (as occurs in some commercial formulations involving multiple EPN species), these signaling mechanisms are muddled.

Movement in different transmission stage(s) is a key aspect in parasite life cycles. Entomopathogenic nematodes have a specialized infective juvenile (IJ) stage, which is mobile, capable of withstanding environmental extremes and equipped to penetrate into the host insect hemocoel. Regardless of whether nematodes are applied to soil as inundative biological control agents, or are fulfilling their role as obligate insect parasites in more natural conditions, the IJ must move through the environment to find and then infect a host. Behaviors related to movement are an important aspect of EPN biology, and understanding how IJs move through space and interact in the soil will provide insights into their ecology and hopefully improve their utility as biological control agents.

In this series of experiments, we measured movement parameters of three species of EPN with different foraging strategies, applied alone or in combination. *Steinernema carpocapsae* has been classified as an ambush strategist that remains near the soil surface and conducts prolonged bouts of foraging by standing on its tail, awaiting passing hosts; *Steinernema glaseri* is a cruise forager that moves through the soil matrix in search of hosts; and *Steinernema feltiae* is intermediate between the other two [1].

Research into EPN movement has begun to explain many of the aspects of their group dynamics. It is well known that EPN populations aggregate due to both abiotic and biotic drivers. They cluster in response to environmental conditions, and their clumped distributions are reinforced by both mass emergence from host cadavers and the need for mass attack to effectively overcome insect defenses. Additionally, EPNs follow the trails of both conspecific and heterospecific IJs, especially in situations where the trial-making nematodes have had direct contact with insect hosts [2]; thus, clusters are also maintained via communication.

While the extent of heterospecific interaction (competition or cooperation) is not entirely understood, it is reasonable to assume that EPNs interact in field conditions. Some researchers have documented cases of niche separation by foraging habit [3], whereas others have found that similarity in host ranges and geography suggest that EPNs may interact in field conditions due to niche overlap [4] [5]. Mixed-species formulations are available commercially as a biological control product, and researchers have applied combinations containing multiple species with varying degrees of success [6] [7] [3] [8].

Previous work has indicated that aggregative behavior is a component of EPN dispersal [9] and that this aggregative pattern is continuous – i.e., that nematodes disperse together after emerging from a host, or are released into the soil from an aqueous solution, rather than dispersing separately and re-aggregating later at some point in space [10]. We investigated how these dynamics are affected when multi-species groups of EPN interact, either when co-applied at a single point or when two populations meet each other. We conducted these analyses in a series of experiments in sand-filled arenas that were maintained in uniform conditions. Because we were particularly interested in whether interspecific avoidance was occurring, we sampled the arena on a more extensive grid in later experiments, modifying protocols from earlier papers such as [9]. This would make it more likely for us to find instances where the species co-occurred in a single patch. Later experiments also focused on how IJs behaved when they were added at opposite corners of the arena, rather than after co-application in the arena center, as an opposite-corner condition would be a more precise way to observe avoidance, as opposed to interspecific differences in dispersal from a common introduction point.

We hypothesized that given the costs associated with mixed-species infections, where even the “winners” of such competitions show reduced fitness in terms of IJ production [2], aggregation should be more intense under mixed-species conditions than in single-species settings. Where two species were co-applied at a single point of introduction, we predicted that if clear species-specific signals enabled IJs to discriminate between species, the aggregations that would be established would be species-specific rather than mixed.

## Materials and Methods

### Entomopathogenic nematodes and experimental arenas

Entomopathogenic nematode species included a cruiser-type forager – *Steinernema glaseri* (VS strain); an ambusher – *Steinernema carpocapsae* (All strain); and an intermediate forager, *Steinernema feltiae* (SN strain). Nematodes were cultured *in vivo* by infecting last-instar *Galleria mellonella*. Infected *G. mellonella* were placed onto White traps [12]. Emerged infective juveniles (IJs) were collected by pouring the contents of the White traps into culture flasks and then diluting them with deionized water to an approximate concentration of 1200 IJs/ml. Flasks were stored at 14°C until used for experiments. IJs for experiments were used within 14 days of emergence.

These experiments were conducted in arenas consisting of boxes fitted with a grid measuring 190mm × 190mm. The boxes were either made of polypropylene for the center application, or Pyrex (Corning Incorporated, Corning, NY) for the opposite-corner application. Boxes were filled with silica sand to a depth of 2.5 cm following previously described methods [9] [10]. The moisture level in the sand was brought to 10% to approximate field capacity with deionized water. Arenas were stored inside plastic bags at 25°C until nematode movement was assessed.

### Single species application (center alone)

**Figure 1:**
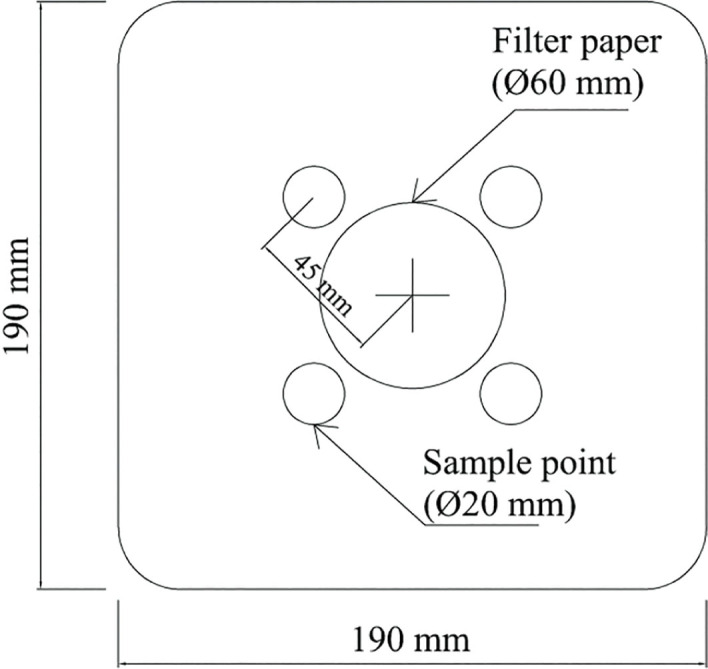
Polypropylene experimental arenas to assess introduction at a common point. Arenas filled with approximately 1200 g of sand at 10% moisture. Image shows introduction point on 60mm filter paper and sample locations.

In single-species trials, nematodes were taken from culture flasks and concentrated onto filter paper using vacuum filtration. A single 60mm diameter filter paper (Whatman No. 1) was used to vacuum the IJs; the filter paper was then placed upside-down in the center of the sand arena (Fig. 1). Approximately 1,200 IJs were applied. Arenas were stored inside plastic bags at 25°C for three days prior to assessment.

After the three-day incubation, filter papers were removed, and sand cores measuring 20 mm in interior diameter and 25 mm in depth were taken at four points located at 45 mm from the center of the arena (diagonally and equidistant from each other, Fig. 1). Nematodes were extracted from the sand in each core, and from the filter paper, by triple-rinsing in deionized water; the number of IJs in each sample was then counted using a stereomicroscope.

### Co-application (center together)

In co-application trials, nematodes were taken from culture flasks and concentrated onto filter paper using vacuum filtration. A single 60-mm-diameter filter paper (Whatman No. 1) was used to vacuum the IJs of both species; the filter paper was then placed upside-down in the center of the sand arena (Fig. 1). Arenas were stored inside plastic bags at 25°C for three days prior to assessment.

After the 3-day incubation, filter papers were removed, and 25-mm deep sand cores with an interior diameter of 20 mm were taken at four points 45 mm from the center of the arena (diagonally and equidistant from each other, Fig. 1). Nematodes were extracted from the sand in each core and from the filter paper by triple-rinsing in deionized water; the precise number of IJs of each species in each sample was then counted using a stereomicroscope.

### Separate corner application (alone and together)

**Figure 2:**
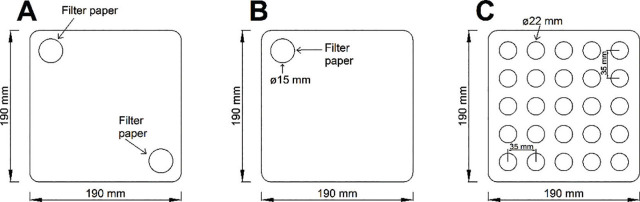
Pyrex experimental arenas to assess responses when nematodes were added to opposite corners. Arenas filled with approximately 1200 g of sand at 10% moisture. A: Heterospecific experiment arena, where corners have different species of IJs. B: Conspecific test arena, where a single species of IJ was placed at one corner. C: 5 × 5 sampling grid; samples were collected at each circle.

Nematodes were applied to separate-corner experimental arenas using two approaches: two different species placed at diagonal corners (Fig. 2A) and a single species at one corner (Fig. 2B). For the application of all treatments, 10,000 IJs from culture flasks were vacuum filtered onto 15-mm-diam. P8 filter paper (Fisher Scientific, Pittsburgh, PA). The filter papers were then placed upside-down in the corners of a sand arena, with nematodes in contact with the sand. Arenas were stored inside plastic bags at 25°C for five days. This extended period was chosen after experimentation because it allowed for IJs under one-corner treatments sufficient time to spread out across more than half of the arena, and time for possible interactions during heterospecific treatments.

After the 5-day incubation, filter papers were removed, and 11-mm-diam. interior sand cores with 25 mm depth were taken on a 25-point grid at points 35 mm from the center (Fig. 2C). Nematodes were extracted from the sand in each core and from the filter paper by triple-rinsing in deionized water; the precise number of IJs in each sample was then counted using a stereomicroscope.

### Experimental design and sample sizes

For experiments conducted with nematodes that were applied to the center of the arena, four replicated dishes were tested and the entire experiment was conducted twice. In assays where applications were in one or more corners, each assay was conducted in time either three times (heterospecific two-corner assay) or four times (single-species single-corner assay).

### Statistical analyses

#### Center-placed nematodes

The Index of Dispersion (D) was calculated in each of the center-placed nematode experiments. In these cases, as in previous experiments [9], [10], the null hypothesis we utilized predicted that nematodes would move independently, with an alternate hypothesis that they would tend to aggregate. We used a single approach for analyzing aggregation in single-species arenas, and two different approaches for assessing aggregation when species were added to the center together. In all cases, the Index of Dispersion [11] [9] was calculated as the ratio of the variance to the mean (D = σ^2^/µ) using the four equal-sized cores that were distributed around the introduction point (Fig. 1). In these cases, an index greater than 1 indicates aggregation, while an index lower than 1 indicates a more uniform distribution than expected given the null hypothesis. We tested whether the index of dispersion was significantly greater than 1 for a given species using a randomization test based on the sum of the indexes of dispersion across replicates [9].

#### Corner placed nematodes - randomization test for aggregation

In the corner-placed experiments, a null hypothesis of no aggregation predicted that expected counts would be the same for points equidistant from the origin. In the square grid layout used in the corner-placed nematode trials, there are two points at each distance from the origin. Since the grid is 5 × 5, this means that there are ten distances and ten corresponding pairs, excluding the diagonal.

According to the hypothesis, if the IJs did not aggregate, then the counts would tend to be similar between points in these pairs. If the IJs did aggregate, then there would tend to be larger differences in counts between points in the pairs. We used a test statistic that quantifies the degree of difference in count between paired points:

$$T=\frac{1}{n}\sum_{i=1}^{n}\frac{\mathrm{var}(x_{ij})}{\mathrm{mean}(x_{ij})}=\frac{1}{n}\sum_{i=1}^{n}\frac{{\left(x_{i1}-x_{i2}\right)}^{2}}{\left(x_{i1}+x_{i2}\right)}$$

Where *i* is calculated over the ten pairs and x_i1_ and x_i2_ are counts of the paired points. This statistic is the average of the index of dispersion over the ten distances, and we report this as the index of dispersion in the results. Because the mean counts vary widely with distance from the release point, calculating the index of dispersion simultaneously over the whole grid would give inflated values.

Calculating *p*-values for this statistic requires a fairly restrictive assumption about nematode movement. If nematodes disperse via independent diffusive movement, then the counts in each core will follow a Poisson distribution. Thus, because the two cores in a pair are at the same distance from the origin, then these Poisson distributions will have the same mean. In this case:

$$E\left(\frac{{\left(x_{i1}-x_{i2}\right)}^{2}}{\left(x_{i1}+x_{i2}\right)}\right)\approx 1$$

The expected value of T in this context is thus equal to 1. The following procedure was used to calculate *p*-values:

[1] We calculated the observed test statistic *T*;
[2] We estimated λ(d), which is the expected count in a core at distance *d* from the origin. This is estimated using locally estimated scatterplot smoothing (LOESS) regression of counts of distance. LOESS regression is a nonparametric regression method that does not depend on a model. It flexibly fits the curve to the shape of the data.
[3] For each pair of off-diagonal points, we generated counts from Poisson λ(d). From these pairs of counts, we then calculated the test statistic *T*.
[4] This third step was repeated 1000 times, and the test statistic values were saved, giving us a null hypothesis distribution for the test statistic.
[5] Finally, we calculated the *p*-value by comparing the observed test statistic to the values from step 4.

### Testing for differences in aggregation

We tested for differences in aggregation behavior between single-species and mixed-species assays in both center-placed and corner-placed experiments. If IJs moved via independent diffusive movement with no aggregation, then the counts in the cores would follow a Poisson distribution. One property of the Poisson distribution is that its mean is equal to its variance. If IJs aggregate, then we would expect to see that the variance in counts between cores is higher than the mean.

The negative binomial distribution is commonly used to model aggregated counts. This distribution has two parameters: the mean *μ* and the aggregation parameter *α*. The variance of the negative binomial is equal to *μ* + α^*^ *μ*^2^. When *α* = 0, then the negative binomial reduces to the Poisson distribution. If *α* > 0, then there is aggregation. The larger the value of *α*, then the stronger the aggregation. We can quantify the degree of aggregation by modeling the data with a negative binomial distribution and estimating the aggregation parameter.

The parameters of the negative binomial were estimated using the maximum likelihood for each assay. The mean parameters were estimated separately for each run, while the aggregation parameter was estimated jointly across all runs in an assay. We assumed that the mean count was determined by distance from the release point. For the center-placed nematodes, all cores were at the same distance. Thus, we estimated a single mean parameter for each run by maximum likelihood. For the corner-placed nematodes, the counts were on a grid and thus were at varying distances from the release point.

There was not enough data at each distance to reliably estimate mean parameters. Instead, LOESS regression was used to combine data across distances and estimate an overall-mean-count-versus-distance function, as described in the previous section. The mean value from this estimate was used in the likelihood function.

For each species, a likelihood ratio test was conducted to determine whether the aggregation parameter was higher when each species was paired with each other species than when a species was isolated. Under the null hypothesis, the aggregation parameter was the same between the two conditions; under the alternate hypothesis, the aggregation parameter was fit separately for each condition. Thus, there was a difference of one free parameter, and the likelihood ratio follows a chi-squared distribution with one degree of freedom.

Finally, we used Fisher’s method to combine *p*-values across corner-placed trials to determine whether IJs tended to aggregate more in the presence of another species than when a species was alone. Note that the index of dispersion can be estimated as $1+\frac{\alpha }{\mu }$ , and thus testing for differences in the aggregation parameter *α* is equivalent to testing for differences in the index of dispersion, after accounting for differences in the mean parameter *μ*.

## Results

### Center-placed nematodes (single and mixed)

**Figure 3:**
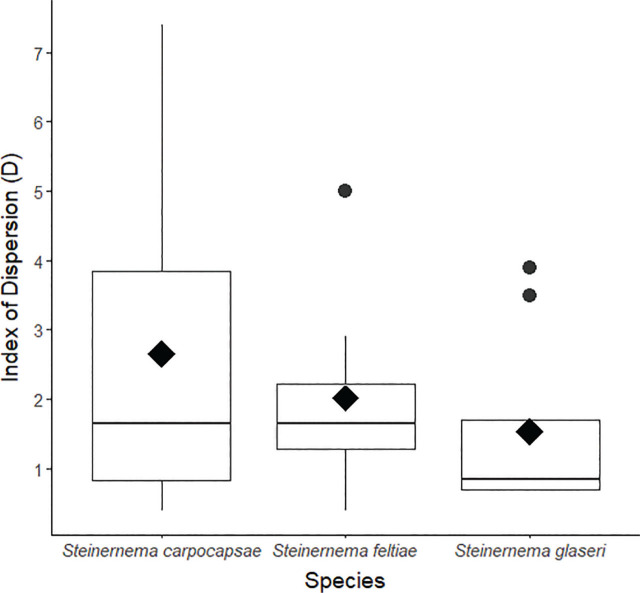
Index of Dispersion for each of the three species when applied alone in the center of dispersal boxes. Values of D > 1 indicate increasing aggregation. Solid line within each box indicates the median, black diamonds indicate the arithmetic mean, and black circles indicate outliers (observations with values > 1.5 * the interquartile range).

In the individual-species experiments, each of the three species showed strong evidence for aggregation (all *P* < 0.05, Fig. 3). This aggregation was statistically significant, despite high levels of variability among trials within each species.

**Figure 4:**
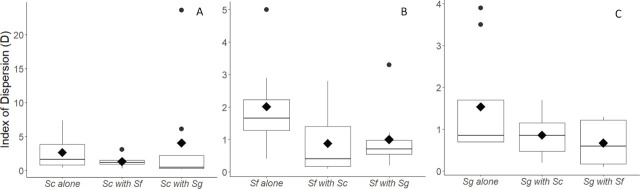
Aggregation shown by each of the three species when center placed, in both conspecific (alone) and heterospecific conditions. Increasing values of D indicate increasing aggregation. Sc = *Steinernema carpocapsae*, Sf = *Steinernema feltiae*, Sg = *Steinernema glaseri*. The solid line within each box indicates the median, black diamonds indicate the arithmetic mean, and black circles indicate outliers (observations with values > 1.5 * the interquartile range). NOTE that y-axis scale changes significantly across the three panels.

When species were paired and co-applied to the center of arenas, only *Steinernema carpocapsae* showed evidence of aggregation; this was driven by strong aggregation in two of the eight trials (Fig. 4A). The other six trials showed a much more uniform distribution, as evidenced by the greater relative size of both the inter-quartile range and the lack of whiskers in Figure 4A. Both *Steinernema feltiae* and *Steinernema glaseri* showed less evidence of aggregation when applied in the presence of other species (Figs. 4B and 4C, respectively), though this change was not statistically significant (0.05 < *P* < 0.25).

### Corner-placed nematodes (single species or two species applied in separate corners)

Using Fisher’s method to conduct an overall test of aggregation in single species vs. mixed species assays in corner-placed experiments showed that each species of nematode aggregated more in the presence of another species than when alone (*P* = 0.006). Looking at individual pairings, aggregation was significantly higher at the 0.05 level in two cases: *S. glaseri* aggregated significantly more when it was paired with *S. carpocapsae* than when it was alone (*P* = 0.047), and *S. carpocapsae* aggregated significantly more when it was paired with *S. feltiae* than when it was alone (*P* = 0.009). While none of the other tests were individually significant, the test pairing *S. glaseri* with *S. feltiae* was close to significant (0.104) and five of six tests had *p*-values of 0.25 or lower.

**Figure 5:**
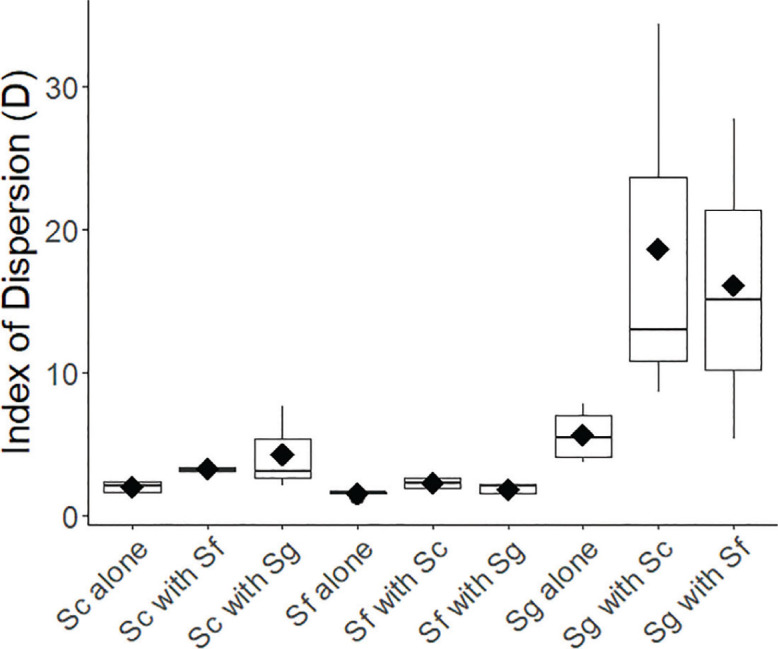
Aggregation shown by each of the 3 species when corner placed, in both conspecific and heterospecific conditions. Increasing values of D indicate increasing aggregation. Sc = *Steinernema carpocapsae*, Sf = *Steinernema feltiae*, Sg = *Steinernema glaseri*. The solid line within each box indicates the median, black diamonds indicate the arithmetic mean, and black circles indicate outliers (observations with values > 1.5 * the interquartile range).

**Figure 6:**
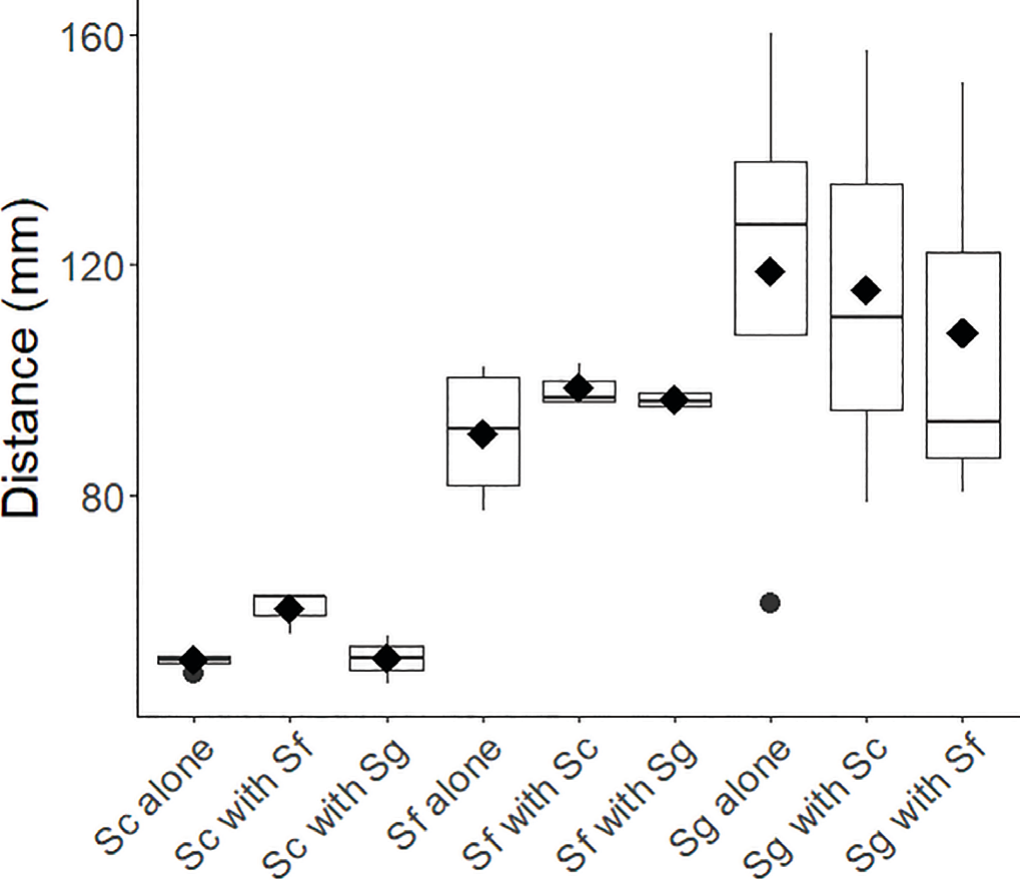
Average IJ movement in each of the 3 species when corner placed, in both conspecific and heterospecific conditions. Sc = *Steinernema carpocapsae*, Sf = *Steinernema feltiae*, Sg = *Steinernema glaseri*. The solid line within each box indicates the median, black diamonds indicate the arithmetic mean, and black circles indicate outliers (observations with values > 1.5 * the interquartile range).

*Steinernema carpocapsae* showed an overall aggregative behavior, demonstrating statistically significant (*α* < 0.05) aggregation in two of four replicates when alone, and in all runs when paired with another species. *S. carpocapsae* also exhibited nearly twice the level of aggregation when paired with other species (mean aggregation statistic of 3.7 ± 0.8) than when alone (1.9 ± 0.3), a difference that was statistically significant (Fig. 5). *S. carpocapsae* showed the least movement (average distance 54 mm), and movement did not differ between single- and mixed-species arenas (Fig. 6).

In general, *Steinernema feltiae* showed less evidence of aggregation across the trials than the other two species (Fig. 5). When alone, the aggregation statistic was only statistically different from 1 in one of the four trials (all *P* ≥ 0.05), averaging 1.5 ± 0.1. The aggregation statistic increased when *S. feltiae* shared arenas with other species; the *S. feltiae* aggregation statistic in mixed arenas averaged 2.0 ± 0.3, and four of the six mixed trials showed statistically significant aggregation (all *P* ≤ 0.02). That said, the increase in the aggregation statistic was only 30%, a much weaker trend than was observed with *S. carpocapsae* or *S. glaseri*, where the increases were approximately 94% and 200%, respectively. *S. feltiae* showed an intermediate level of movement (average distance 95 mm), with no difference in movement between single- and mixed-species arenas (Fig. 6).

*Steinernema glaseri* aggregated more strongly than the other two EPN species, whether by itself or when paired with other species (all *P* < 0.01, Fig. 5). The average aggregation statistic was higher when *S. glaseri* was paired with other species (mean aggregation statistic of 17.3 ± 4.6) than when by itself (5.6 ± 1.0). *S. glaseri* exhibited the most movement of the three species (average distance 114 mm), though there were no differences in the distance moved between single- and mixed-species trials (Fig. 6).

## Discussion

While prior research has shown that EPN display aggregative movement [9] [10], here we show that movement patterns are altered by the presence of other EPN species and the manner in which the EPN species are exposed to each other. Support for our initial hypothesis that aggregation should be more intense under mixed-species conditions than single-species settings depended on context. Where nematodes were co-applied in the center of arenas, we saw that EPN tended to aggregate less than they would when applied alone. In corner applications, where the nematodes started off at opposite sides of the arena, we saw strong evidence for greater levels of conspecific aggregation in mixed-species vs. single-species arenas, despite similar levels of EPN movement. In these corner-placed trials, then, nematodes moved just as much in mixed-species arenas as they did when alone, but tended to be more aggregated in the presence of another species.

Why did we see such differences between co-applied and corner-applied mixed-species aggregations? We believe this likely has to do with species-level differences in the ascarosides that are produced by these species. Ascarosides are biochemically active glycosides of the dideoxysugar ascarylose; ascaroside molecules play many roles in EPN and other nematodes, including regulating movement behaviors such as dispersal, trail following, etc. Each EPN species appears to produce species-specific blends of common ascarosides, with many differences between species being driven by the ratios among this common suite of chemicals [12]. These would be effective at maintaining species-specific groupings, but when two or more species are artificially mixed together (as we did here for center co-application, or as is done in some commercial formulations containing multiple EPN species), the ascaroside signals that would otherwise facilitate avoidance of heterospecifics and aggregation with conspecifics become muddled.

Wu and Duncan [14] investigated how mixtures of multiple species of EPN affected dispersal, using IJs of *Heterorhabditis indica* co-applied with either *Steinernema disprepesi* or *S. glaseri*. They found that the presence of *S. diaprepesi* significantly increased the dispersal rate of *H. indica*, while the presence of *S. glaseri* did not. While those trials used more distantly-related species than we used (combining heterorhabditids and steinernematids), it still appears that there are differences in interspecific communication among steinernematids, at least as perceived with *H. indica*. Thus, while ascaroside signaling is broadly conserved [15], species-level differences exist, and formulations that involve combining IJs from multiple species and co-applying them may have unpredictable effects due to signaling interference.

While it is possible that compounds other than ascarosides are regulating these behaviors, given the previously-identified role of ascarosides in promoting dispersal [12], aggregation [16] and other social behaviors ([17], [18]), ascarosides seem the most likely suspect. We are presently investigating the chemistry that regulates species-specific aggregation of several species of entomopathogenic nematodes, with the objective of establishing the role of ascarosides in maintaining species-specific aggregative behaviors.

The results from the corner-placed portion of the experiments complement recent work on group behaviors in EPN. In tests of group joining behavior using six-arm olfactometers, *S. glaseri* IJs sometimes moved towards (“joined”) other species of EPN; however, they were only inclined to join groups of *S. feltiae* IJs that had experienced recent (~ 24-hr prior) contact with a host. *S. glaseri* did not appear inclined to join groups of *S. feltiae* IJs that had not experienced host contact, and were not attracted to groups of *S. carpocapsae* IJs in any context [19].

Extending those results to what we observed in the corner-placed trials suggests that without specific cues related to host presence, EPN IJs tend to avoid each other. This is supported by recent work on following behavior among these same three species, where there was no tendency for individual IJs to follow trails made by heterospecific IJs unless the “leader” on the trail had had prior contact with the host [2].

Scaling the avoidance behavior we observed in the corner-placed trials up to field conditions, it may hold the potential to mitigate interspecific competition and thereby promote diversity within the entomopathogenic nematode community. Many field surveys have found that multiple EPN species co-occur within fairly small areas [20], [21], [22]. While multiple EPN species can co-infect a host, there are costs to these co-infections, as early invaders may be attacked and killed by heterospecific or conspecific males [23], and even if one species succeeds within the host, there is generally a cost associated with this competition ([6] [24] [22] [2]). While niche separation based on factors such as foraging strategies, host specialization and depth preferences [25] [6] [4] certainly play a role in maintaining diversity, we show here that EPN appear to increase the level of conspecific aggregation in mixed-species environments.

It is important to consider that the current experiment used IJs stored in water prior to application, rather using IJs that emerged directly from cadavers into the arenas. Shapiro-Ilan et al. [9] compared aggregation behavior among six species in single-species arenas and found only one significant difference in aggregation behavior between aqueous-stored and cadaver-emerged IJs. In those trials, *S. glaseri* IJs were more homogenously distributed (i.e., they did not demonstrate aggregation) when they had emerged directly from cadavers. IJs that emerge directly from cadavers have been shown to demonstrate increased dispersal and infectivity [26], and exposing aqueous-stored IJs to cadaver-derived extracts has been shown to cause those IJs to demonstrate increased dispersal and infectivity [27], [28]. It is unclear whether the aggregation behaviors we observed here would change in natural systems, where IJs emerge from insect cadavers into the soil; this may be an appropriate next step in investigating this phenomenon.
